# Peptide Hydrogelation and Cell Encapsulation for 3D Culture of MCF-7 Breast Cancer Cells

**DOI:** 10.1371/journal.pone.0059482

**Published:** 2013-03-20

**Authors:** Hongzhou Huang, Ying Ding, Xiuzhi S. Sun, Thu A. Nguyen

**Affiliations:** 1 Department of Grain Science and Industry, Kansas State University, Manhattan, Kansas, United States of America; 2 Department of Biochemistry, Kansas State University, Manhattan, Kansas, United States of America; 3 Department of Diagnostic Medicine/Pathobiology, Kansas State University, Manhattan, Kansas, United States of America; University of California, San Diego, United States of America

## Abstract

Three-dimensional (3D) cell culture plays an invaluable role in tumor biology by providing *in vivo* like microenviroment and responses to therapeutic agents. Among many established 3D scaffolds, hydrogels demonstrate a distinct property as matrics for 3D cell culture. Most of the existing pre-gel solutions are limited under physiological conditions such as undesirable pH or temperature. Here, we report a peptide hydrogel that shows superior physiological properties as an *in vitro* matrix for 3D cell culture. The 3D matrix can be accomplished by mixing a self-assembling peptide directly with a cell culture medium without any pH or temperature adjustment. Results of dynamic rheological studies showed that this hydrogel can be delivered multiple times via pipetting without permanently destroying the hydrogel architecture, indicating the deformability and remodeling ability of the hydrogel. Human epithelial cancer cells, MCF-7, are encapsulated homogeneously in the hydrogel matrix during hydrogelation. Compared with two-dimensional (2D) monolayer culture, cells residing in the hydrogel matrix grow as tumor-like clusters in 3D formation. Relevant parameters related to cell morphology, survival, proliferation, and apoptosis were analyzed using MCF-7 cells in 3D hydrogels. Interestingly, treatment of cisplatin, an anti-cancer drug, can cause a significant decrease of cell viability of MCF-7 clusters in hydrogels. The responses to cisplatin were dose- and time-dependent, indicating the potential usage of hydrogels for drug testing. Results of confocal microscopy and Western blotting showed that cells isolated from hydrogels are suitable for downstream proteomic analysis. The results provided evidence that this peptide hydrogel is a promising 3D cell culture material for drug testing.

## Introduction

Two-dimensional (2D) substrates, such as tissue culture polystyrene and the surface of tissue analogs, make an enormous contribution to modern *in vitro* cell studies; however, traditional 2D platforms can not accurately mimic the complex 3D architecture of the extracellular matrix (ECM) where native cells reside [1]–[4]. In 2D culture, the monolayer cells experience homogenous concentration of nutrients and growth factors which induce unnatural cell environments and cell-cell interactions, yielding a flat and stretched morphology [5]. Recent studies have shown that the morphological differences of cells cultured in 2D and 3D can exhibit several striking differences in subtle cellular processes such as proliferation, apoptosis, differentiation, gene expression, migration, and drug sensitivities [6]–[9]. On the other hand, the biological *in vivo* 3D systems, such as animal models, are expensive and time-consuming. Therefore, advanced *in vitro* 3D model systems are needed to fill the gap between the inaccurate 2D systems and the animal models, mimicking the complexity of the ECM and the physiological relevance of an *in vivo* biological system.

In the last few decades, hydrogel scaffolds, cross-linked networks that possess high water contents, have attracted more and more attention in an attempt to mimic *in vivo* conditions for cell culture. The reticulated structure of cross-linked polymer chains with high water contents introduces a number of desirable cellular microenvironment characteristics: 3D spatial support for cell growth; porosities for cell migration; and facile transportation of oxygen, nutrients, waste, and soluble factors [10]–[16]. Hydrogels can be formed from a range of natural sources and synthetic materials. Natural gels derived from ECM components and other biological sources such as collagen, fibrin, hyaluronic acid, chitosan, and alginate are biocompatible and inherit bioactivities that promote cell survival, proliferation, differentiation, and cellular function of many cell types [17]–[20]. However, natural hydrogels have varying biochemical presentations and material properties that are difficult to control, which increases the risk and complexity of cellular study in this culture system [21]. On the other hand, synthetic gels are highly reproducible with consistent composition and predictable manipulation of properties [22]–[24]. However, synthetic polymers such as polyactide and polyglycolide have too large fiber diameter and porous size, which present poor scaffold structure and mechanical properties to accurately mimic the the full complexity of natural environment of cell growth [21]. With the rapid development of rationally designed peptides as biological materials [25]–[29], peptide based hydrogel was considered as one of the most promising material for 3D cell cutlure because of its amino acid composition and the structural and mechanical similarity to natural ECM [30]–[32].

In addition, for *in vitro* 3D cell culture, cell encapsulation and isolation are two critical steps to introduce 3D spatial support for cell growth and recover embedded cells from scaffold matrix for downstream studies respectively. For a convenient, effective, and safe encapsulation, cells should be added simultaneously with the initialization of hydrogelation [33]–[35]. Therefore, mild and cyto-compatible hydrogel-forming conditions are preferred, to ensure that cells survive comfortably during gel formation. However, the solubility of gel transformation of current peptide/protein hydrogels (i.e., puramatrix gel, hydromatix peptide hydrogel, and matrigel) is triggered by adjusting pH or temperature (Table S1). The undesirable low pH or cold temperature of the pre-gel solutions may cause the cell death when they are directly mixed. Hydrogel preparation procedure become complex when changing cell medium for pH balance and chilling experimental tools are required (Table S1). Cells kept in sucrose solution or a gel-forming buffer struggle with lack of nutrients up to several hours during gel formation before cell medium can be added. Moreover, isolating cell from hydrogel matrix is another challenge to 3D cell culture. For most cases, changing the environmental factors back to extreme conditions or adding undesirable buffer for hydrogel degradation are required to initial the solubility of gel transformation before cells can be separated out (Table S1). This process threatens the survival of cultured cells and may cause the failure for the whole downstream studies. Therefore, it is necessary to develop a hydrogel which not only presents a convenient and effective process for cell encapsulation, but also provides easy and safe cell isolation for further cell physiological and pathophysiological studies.

In this study, a solution of a newly identified peptide called h9e was prepared at neutral pH and mixed with Minimum Essential Medium (MEM, with 10% FBS) at room temperature. After mixing, h9e peptides self-assemble into a hydrogel matrix with a final peptide concentration as low as 1 mM (0.17%). Without introducing any additional gel forming buffer or adjusting environmental pH or temperature, the peptide provides a convenient and mild hydrogel forming process and allows cells to be surrounded by their culture medium during cell encapsulation (Table S1). More interestingly, the mechanical strength of this hydrogel matrix exhibits special deformability and reassembly capability, which allow the gel-soluble transformation through repeated pipetting. A breast cancer cell line, MCF-7, was selected as a model to grow in 3D culture of the h9e-MEM hydrogels. Studies of cell morphology, viability and proliferation showed that cells exhibited 3D cyto-architecture in the hydrogel matrix and kept high bioactivities for further studies after isolation. Cisplatin, an anti-cancer drug, was used to examine its efficacy on MCF-7 cells in hydrogel matrix. Overall, the results show a strong support that the h9e peptide is a promising 3D cell culture material for drug testing.

## Materials and Methods

### Materials

N,N-Dimethylformamide (DMF), Trifluoroacetic acid (TFA), piperidine, N,N-Diisopropylethylamine (DIEA), Triisopropylsilane (TIS), paraformaldehyde, glutaraldehyde, cis-Diamminedichloro-platinum, 0.4% Trypan blue, insulin, and anti-actin antibodies were purchased from Sigma-Aldrich (Milwaukee, WI). N-Methylpyrrolidinone (NMP), anhydrous ether, dichloromethane (DCM), and sodium bicarbonate were purchased from Fisher Scientific (Pittsburgh, PA). Rink Amide MBHA Resin, 2-(1H-Benzotriazole-1-yl)-1,1,3,3-tetramethyluronium Hexafluorophosphate (HBTU), and all protected amino acids were purchased from EMD Biosciences (San Diego, CA). N-hydroxybenzotriazole (HOBT) was purchased from CEM (Matthews, NC). Sodium pyruvate, non-essential amino acids, MEM with Eagle’s salts, TrypLE Express trypsin solution, 4′, 6-diamidino-2-phenylindole (DAPI), and Hoechst 33342 fluorescent dye (10 mg/mL) were purchased from Invitrogen (Carlsbad, CA). Fetal bovine serum (FBS) was purchased from Atlanta Biologicals (Lawrenceville, CA). Anti-survivin, anti-Ki67, and anti-caspase-3 antibodies were obtained from Santa Cruz Biotechnologies (Santa Cruz, CA). Anti-cleaved caspase-3 and HRP-linked anti-rabbit/mouse antibodies were purchased from Cell Signaling Technology (Danvers, MA).

### Cell Culture

The MCF-7 human breast cancer cell line was purchased from American Type Cell Culture (ATCC) (Manassas, MA). The cells were grown in MEM medium with 10% (v/v) FBS, 1 mM sodium pyruvate, 17.9 mM sodium bicarbonate, 0.01 mg/mL insulin, and 1% (v/v) non-essential amino acid. Cells were maintained in T-75 cm^2^ tissue culture flasks (Greiner Bio-one, Begium) at 37°C with 5% (v/v) CO_2_. Cells were routinely passed with trypsin and medium was changed every other day.

### Peptide Synthesis and Hydrogelation

The h9e peptide was synthesized according to a previously published protocol [36]. Briefly, peptides were synthesized on an automated CEM Liberty microwave peptide synthesizer (CEM Corporation, Matthews, NC) according to the base-labile 9-fluorenylmethoxycarbonyl (Fmoc) strategy with Rink amide resin and Fmoc-protected amino acids. After final N-terminal Fmoc group deprotection, the resin-bound peptides were side-chain-deprotected and cleaved using TFA/TIS/water (95/2.5/2.5 v/v). Peptides were precipitated and washed three times with anhydrous ether, dissolved in acetonitrile and deionized water (50/50 v/v), then freeze-dried. Molecular weight and purity of the synthesized peptides were confirmed by matrix-assisted laser desorption/ionization time-of-flight mass spectroscopy and high-performance liquid chromatography.

Lyophilized peptide was added to 100 mM sodium bicarbonate and completely dissolved by magnetic stirring for 3 hours with a final peptide concentration of 10 mM. For hydrogelation, peptide solution was added into MEM with 10% FBS and the mixture was hand-shaken for about 10 seconds. The peptide hydrogel formed within 15 minutes at room temperature with final peptide concentration of 1, 2, and 3 mM.

### Rheological Tests

The storage and loss moduli (G′ and G″, respectively) of h9e hydrogels were determined on a C-VOR 150 rheometer system (Malvern instruments, Malvern, Worcestershire WR141XZ, United Kingdom) with a 20-mm diameter parallel plate geometry and 500 µm gap size. To mimic cell physiological conditions, all rheological tests were performed at 37°C unless otherwise specified. The peptide and MEM mixture was placed on the measuring system immediately after mixing for a gel-forming rate test. Single frequency (1 Hz) and steady shear strain (1%) were selected for a 1hour test. To determine the hydrogel reassembly capability, the peptide and MEM mixture was incubated at room temperature overnight for hydrogelation, then transferred to a lower measuring plate for 10 minutes, single-frequency test (1 Hz, 1% strain) for stabilization. The hydrogel was broken using 1 Hz frequency and 500% shear strain for 1 minute. Afterward, resetting the instrument parameters took 1 minute, and the hydrogel moduli during the reassembly period were measured under 1 Hz frequency and 1% shear strain for 1 hour. The amplitude sweep test (strain from 1 to 500%, 1 Hz frequency) was conducted multiple times to determine hydrogel reassembly capability after every time it was destroyed. Four testing circles were applied in this measurement and the hydrogel recovery time between every two circles was 1, 5, and 10 minutes. Furthermore, to test the response to different environmental temperatures, the peptide hydrogel was measured under a temperature profile test with steady oscillatory frequency (1 Hz) and strain (1%). The temperature was adjusted from 4°C to 50°C for two testing circles. For each circle, the instrument’s heating or cooling processes took 5 minutes, then another 5 minutes to arrive at the setting temperature (4°C or 50°C). To determine the G′ and G″ of hydrogel during cell isolation, 3 mM peptide hydrogel was diluted 15 times with MEM. After thorough mixing, the diluted solution was tested under 1 Hz frequency and 1% shear strain at 4°C for 1 hour.

### Culturing Cells in h9e Hydrogel

The h9e solution was sterilized by UV irradiation (254 nm) for 30 minutes before cell encapsulation. MCF-7 cells were detached from the flask using trypsin and pelleted by centrifugation at 2,000 rpm for 5 minutes. The supernatant was removed and pelleted cells were re-suspended in MEM. The number of cells was counted using a cellometer Auto 2000 (Nexcelom Bioscience, Lawrence, MA). MEM containing MCF-7 cells were thoroughly mixed with h9e peptide solution and the mixture was seeded into each well of a 12-well culture plate (Becton Dickinson Labware, Franklin Lakes, NJ). The culture plate was placed in a 37°C incubator (Thermo Scientific, Asheville, NC) for approximately 30 minutes. After the complete hydrogelation, 1 mL of MEM was carefully added to the top of the hydrogel to prevent drying during long-term incubation. The medium on the top of the hydrogel was changed every 2 days.

### Cell Isolation from h9e Hydrogel

To isolate the cells from the hydrogel matrix, 2 mL MEM was added to each hydrogel cell culture in the 12-well plate. The hydrogel with embedded cells was thoroughly mixed with MEM using a pipette and transferred to a centrifuge tube. An additional 4 mL MEM was added to wash the cell culture well and collected in the centrifuge tube. One mL trypsin solution was added to the well, which allowed cells to detach from the well. The plate was incubated at 37°C for 5 minutes and the solution was collected into the centrifuge tube again. The centrifuge tube was placed on ice. Another 6 mL MEM was added to the centrifuge tube for a final volume of 15 mL. The solution was thoroughly mixed and cells were pelleted by centrifugation at 2,000 rpm for 5 minutes at 4°C. The supernatant was removed and the cell pellet was collected.

### Cell Distribution Assay

MCF-7 cells (1 × 10^6^ cells/well) were cultured in 3 mM h9e/MEM hydrogel for 5 days. The cell clusters were isolated from the gel matrix and re-suspended in 2 mL MEM. Ten µL (10 mg/mL) Hoechst 33342 fluorescent dye was added to stain the cell nuclei. The cell solution was incubated at 37°C for 30 minutes. Labeled cells were washed for 3 times with MEM medium and re-capsulated within the 3 mM h9e/MEM hydrogel matrix. The cell/gel constructs were observed on a LSM 700 confocal microscope (Zeiss, Jena, Germany).

### Scanning Electron Microscopy (SEM)

The nanofiber network of hydrogel scaffolds as well as surface characters of the 3D cultured cells were observed under SEM. The hydrogel samples were dehydrated with increasing concentrations of ethanol from 50% (v/v) to 100% (v/v) at 5% per step and 15 minutes for each step. The ethanol was then removed by a critical point dryer (Samdri-790B, Tousimis Research Corp., Rockville, MD). The hydrogel samples with cells were fixed in a 2% paraformaldehyde and 2% glutaraldehyde mixture for 30 minutes before dehydration and critical point drying. Samples were then sputter-coated (Desk II Sputter/etch Unit, Denton Vaccum, Moorestown, NJ) 3 times (12 seconds each time) with 100% Pt. The SEM observation was carried out with an FEI, Nova NanoSEM 430 (Hillsboro, ON) at 5 kV and through a lens detector.

### Cell Morphology

MCF-7 cells (2.8×10^5^ cells/well) were cultured in 2D monolayers or 3D hydrogels (1, 2 and 3 mM h9e/MEM hydrogel) for 1, 3, 5, and 7 days. The morphological characters of the cells were determined using an inverted light microscope (Nikon Eclipse TE2000-u, Kanagawa, Japan) at days 1, 3, 5, and 7.

### Cell Viability and Cell Proliferation

MCF-7 cells (2.8×10^5^ cells/well) were cultured in 2D monolayers or 3D hydrogels (1, 2 and 3 mM h9e/MEM hydrogel). Cells in 2D culture were harvested and cells in 3D culture were isolated at days 1, 3, 5, and 7. A cell suspension was mixed with 0.4% trypan blue dye at 1∶1 (v/v) ratio. Cell viability and the number of viable cells were examined by Cellometer Auto 2000 (Nexcelom Bioscience, Lawrence, MA).

### Treating Cells with Drugs

MCF-7 cells (0.5×10^6^ cells/well) were cultured in 3 mM h9e/MEM hydrogel for 5 days. After 5 days incubation, cell spheroids were isolated and treated with 40 µM cisplatin via three methods: top surface, top-bottom transwell, and pre-mixed with medium and peptide. For the top surface method, isolated cell spheroids were re-capsulated within the 3 mM h9e/MEM hydrogel matrix and cultured in 12-well plates. One mL of MEM medium containing 40 µM cisplatin was placed on top of the hydrogel in each well. For the transwell method, isolated cell clusters were re-capsulated within the 3 mM h9e/MEM hydrogel matrix and cultured on a transwell insert. MEM medium containing 40 µM cisplatin was placed on top and bottom chambers. For the pre-mixed method, isolated cell clusters were resuspended in MEM medium containing 3 mM h9e peptide and 40 µM cisplatin. The mixture was added in 12-well plates (1 mL of mixture/well) for hydrogelation. One mL of MEM was carefully added to the top of the hydrogel to prevent drying during long-term incubation. Medium was changed every 2 days in all the methods. Cell viability was examined at days 0, 1, 3, 5, and 10 by using the trypan blue excision method.

### Immunofluorescence and Confocal Microscopy

MCF-7 cells were cultured on coverslips for 2D monolayer culture and with hydrogels for 3D culture. Cells were treated with cisplatin according to each condition, 2D or 3D cultures. Cell clusters in hydrogel culture were isolated. Cells grown on the coverslips or isolated cell clusters were fixed in 4% formaldehyde in PBS for 30 minutes. Cells were washed with PBS and then treated with 0.1% Triton-X 100 for 8 minutes for 2D monolayer culture and 1 hour for the 3D hydrogel culture. Cells were washed with PBS and blocked with 3% bovine serum albumin (BSA) in PBS for 1 hour at room temperature. After blocking, cells were incubated with primary antibodies overnight at 4°C. For the clusters of cell from 3D hydrogel culture, exposure to primary antibodies occurred in 1.5 mL tubes on an orbital rocker. Cells were incubated with Alexa-conjugated secondary antibodies for 1–6 hours at room temperature. DAPI was used to stain nuclei. The coverslips were mounted and sealed on histological glass slides with prolong-antifade reagent (Invitrogen, Camarillo, CA). To avoid flattening of cell clusters, a spacer was used to create a space between the coverslip and microscope slide. Image was captured using a confocal microscope (Carl Zeiss LSM 700 META, Narashige, MN). For the clusters, optical slices were obtained at adequate intervals on the Z axis (between 0.5 and 1 µm). Z-stack images were reconstituted in ZEN 2010 software.

### Western Blot Analysis

MCF-7 cells, cultured in 2D monolayer and 3D hydrogel, were treated with cisplatin. Cells grown in 2D were trypsinized and pelleted by centrifugation at 2,000 rpm for 5 minutes. Cells grown in 3D hydrogels were isolated as described above. After washing with PBS, cells were incubated in lysis buffer (Cell Signaling Technology, Danver, MA) for 5 minutes. Cell lysates were sonicated using Vibra-Cell sonicator (Sonics & Materials Inc, Danbury, CT) and then centrifuged at 13,000 rpm for 30 minutes at 4°C. After centrifugation, supernatants were collected as whole cell extracts. Thirty ug of samples were separated by 4–20% gradient SDS-PAGE for 35 minutes at 200 V, and transferred to nitrocellulose membranes (Midwest Scientific, Saint Louis, MO). Membranes were blocked with 5% milk for 30 minutes and immunoblotted against protein of interest. Immunoreactions using chemiluminescence were visualized by FluorChem E Imaging Instrument (ProteinSimple, Santa Clara, CA). Intensities of the bands were digitized using Un-Scan-It software.

### Statistical Analysis

Two-sample t-test was used to analyze the difference among the various treatments. P-value of less than 0.05 was considered statistically significant.

## Results and Discussion

### Peptide Hydrogelation in MEM

**Figure 1:**
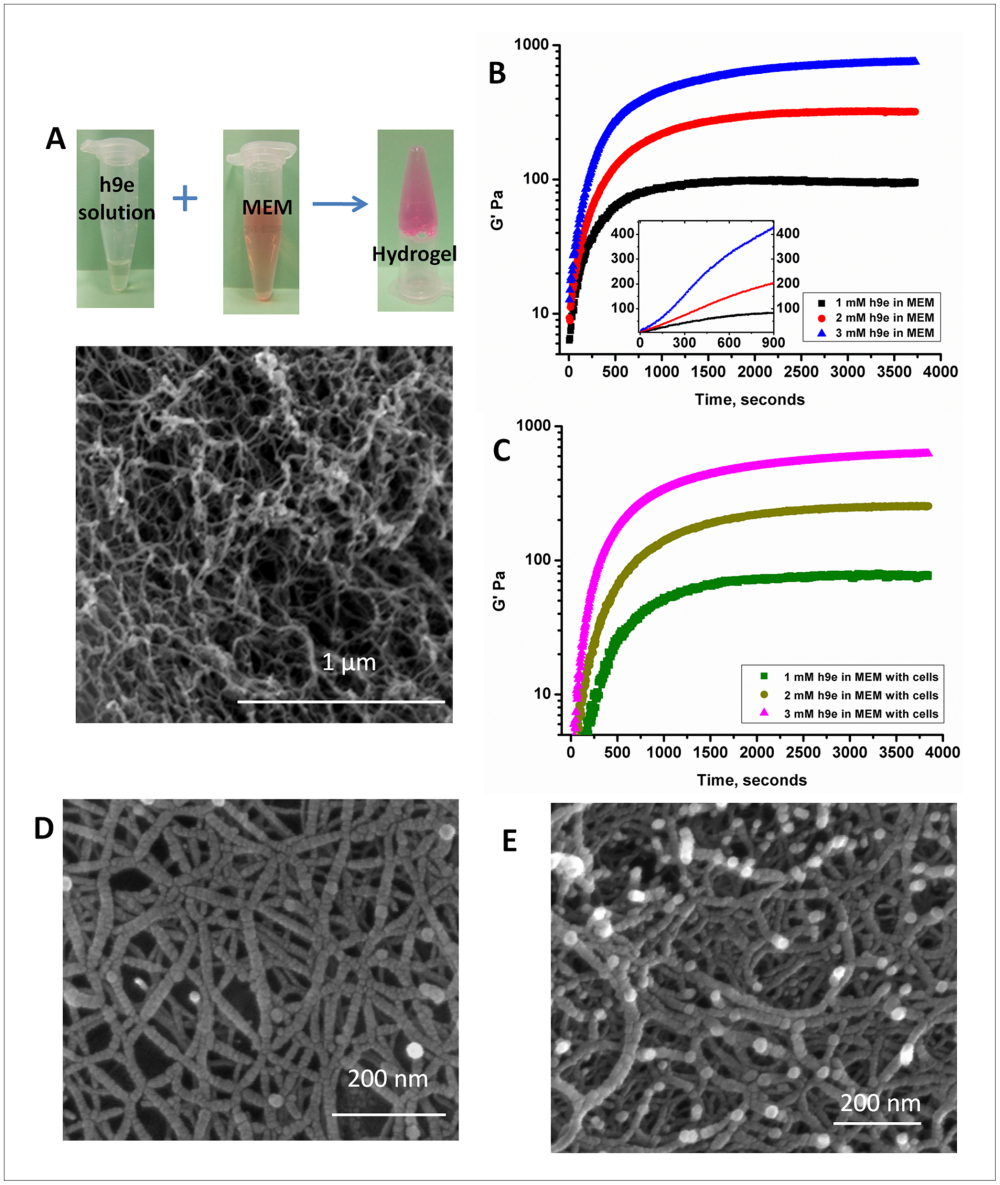
Peptide hydrogelation in MEM. **A.** Proposed mechanism of MEM-induced h9e peptide self-assembling hydrogelation (SEM image showing the nanofiber scaffold of the hydrogel matrix). **B.** Storage modulus G′ of 1, 2, and 3 mM peptide hydrogel during the hydrogelation at 37°C. **C.** SEM image of 1 mM peptide hydrogel. **D.** SEM image of 3 mM peptide hydrogel.

To initiate gel formation, 100 µl of 10 mM h9e peptide solution (pH 7–8) was added to 900 µL MEM medium to form 1 mL mixture with 1 mM (0.17% w/v) peptide concentration (Figure 1A). The nanoscale morphology of the hydrogel matrix is presented by the SEM image (Figure 1A). Peptide hydrogelation induced directly by mixing neutral pH peptide solution with MEM not only avoids the complex chemical gel cross-linking processes, but also utilizes a medium commonly used in biological and medical research, providing a physiological condition to cell encapsulation.

Direct loading of drugs, proteins, or cells during gel formation is one of the most convenient and effective ways for encapsulation [33], [34]. To ensure homogenous distribution of embedded molecules, peptides should assemble as a nanofiber network in a relatively short period with reasonable strength, holding the suspended molecules before their precipitation. To determine the peptide gel formation rate, hydrogels were prepared with three concentrations, 1 mM (0.17% w/v), 2 mM (0.34% w/v), and 3 mM (0.51% w/v), in MEM. The storage modulus of the solution was measured at 37°C immediately after thorough mixing. Figure 1B shows the h9e peptide hydrogel formations with stable storage modules around 100, 400, and 700 Pa, respectively. The gel formation rates increase with peptide concentrations (inset of Figure 1B), and all three hydrogels reached a self-supporting strength (close or above 100 Pa) within 15 minutes. SEM images (Figure 1C, D) indicate that the hydrogel architecture is built by entanglement of 20 nm width nanofibers; however, the lower-concentration hydrogel (1 mM, Figure 1C) shows a relatively looser matrix structure compared with the compact structure of the higher concentration hydrogel (3 mM Figure 1D). This visual evidence further supports the strength differences of different concentration hydrogels.

### Dynamic Rheological Study of h9e Hydrogel

**Figure 2:**
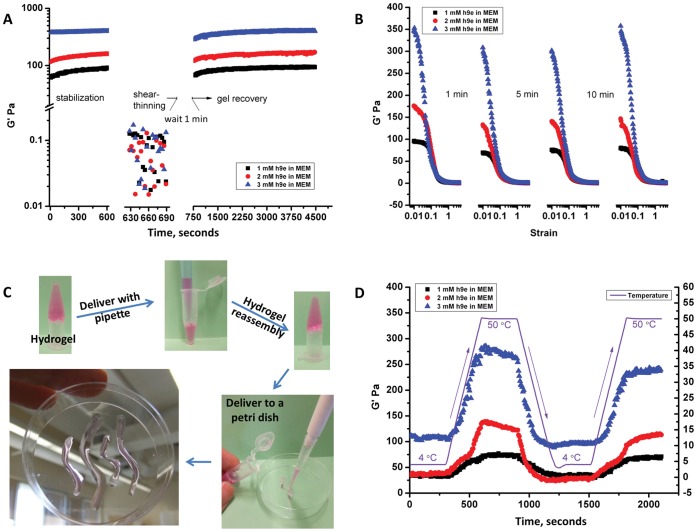
Dynamic rheological study of h9e hydrogel. **A.** Storage modulus G′ of shear-thinning and recovery test of 1, 2, and 3 mM peptide hydrogel. **B.** Four times amplitude sweep test with shear strain from 1% to 500% and 1- 5-, and 10-minute breaks. **C.** Multiple times delivery of peptide hydrogel via pipette; hydrogel was shear thinning but reassembled quickly without permanently destroying hydrogel architecture. **D.** Temperature profile test of 1, 2, and 3 mM peptide hydrogel between 4°C and 50°C.

The deformability and reassembly ability of MEM-induced h9e hydrogel were assessed by a dynamic rheological test. Peptide hydrogels with 1–3 mM were stored at room temperature overnight, then transferred to measuring system and stabilized for 10 minutes. By shear-thinning at 500% strain for 1 minute, all three hydrogels were converted to liquid state, showing a G′ lower than 0.2 Pa (Figure 2A). After shear-thinning stopped, instrument parameters were reset after 1-minute waiting time and the hydrogel recovery was monitored using 1% shear strain for 1 hour. The data in Figure 2A the G′ of hydrogel recovery during this 1 hour test.

To determine whether the hydrogel could maintain the reassembly capability even after shear-thinning many times, the hydrogel was measured under an amplitude sweep test conducted multiple times. Four testing circles were applied in this measurement and shear strain was increased from 1% to 500% within 5 minutes for each circle. For hydrogel recovery, the waiting time of 1, 5, and 10 minutes were applied, respectively. Figure 2B suggests that although the hydrogel architecture was completely broken into liquid form at the end of each circle, quick reassembly persisted even after shear-thinning multiple times. The results also showed that the percentage of recovery G′ increased with waiting time and that the hydrogel reassembly rate related to hydrogel concentrations. For example, with waiting time of 1 minute, about 73%, 76%, and 88% of the gel strengths were recovered for 1, 2, and 3 mM hydrogel, respectively, but after three shear-thinning circles and 10 minutes of waiting, 83% (1 mM), 84% (2 mM), and 100% (3 mM) of the gel strength were recovered (Figure 2B). The higher reassembly rate is most likely caused by the more compact matrix structure of hydrogel due to higher peptide concentration (3 mM) (Figure 1D). In the solution with high concentration of peptide, some non-covalent gel network cross-links remain intact and the broken nanofiber groups are close to each other, making rebuilding the cross-links easy [37]. Based on these rheological properties, the MEM-induced h9e hydrogel could be delivered via pipetting multiple times without permanently destroying the hydrogel architecture (Figure 2C). This special shear-thinning and recovery properity of the hydrogel also provides an alternating method for cell isolation from hydrogel matrix through a mechanical shearing and dilution.

For biological study, temperatures between 4°C and 37°C are commonly applied for many standard operational procedures *in vitro*; therefore, the response of the hydrogel materials to the temperature variations has a large impact on their practical applications. The rheological temperature profile test was performed to address this challenge. The temperature was adjusted from 4°C to 50°C for two testing circles. Figure 2D presents that the G′ of hydrogels moves along with temperature and performs 2–3 times higher at 50°C than that at 4°C. This thermal response is reversible according to the hydrogel heating and cooling circles (Figure 2D). The results provide evidence for using h9e peptide hydrogel as a 3D cell culture. The hydrogel matrix is stiffened for cell encapsulation when it remains at 37°C, but is weakened at 4°C for cell isolation using standard centrifuge method.

### Cell Distribution and 3D Cells Culture in h9e Hydrogel

**Figure 3:**
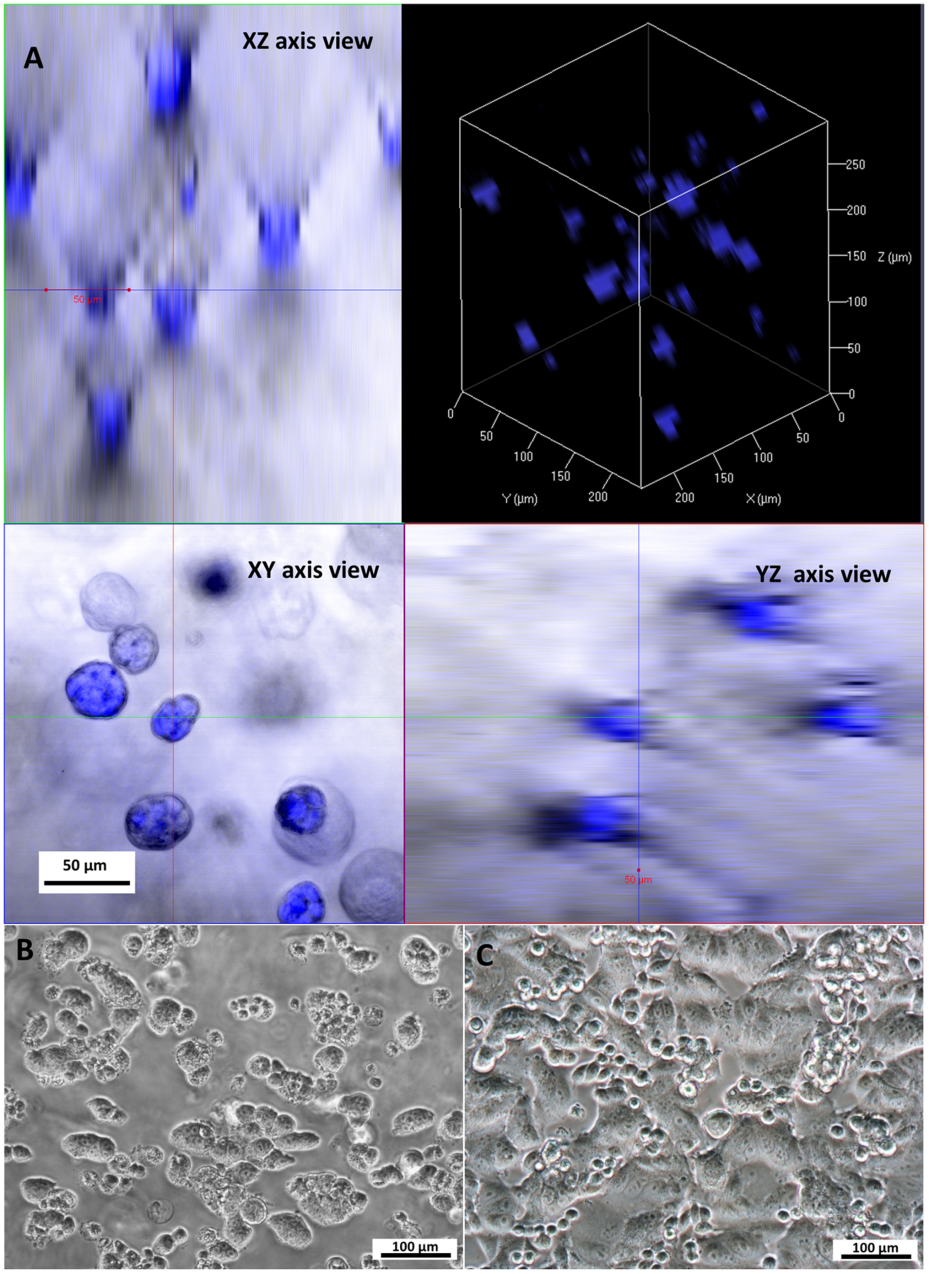
Culturing cells in h9e hydrogel. **A.** LSCM image of cells distribution within hydrogel architecture. **B.** Morphology of cluster cell in 3D hydrogel matrix over 5 days. **C.** Morphology of cells growth on 2D plastic monolayer over 5 days.

The 3D cell culture matrices were prepared by directly adding peptide solution into MCF-7 cell suspension. The cells were encapsulated within the hydrogel scaffold during the peptide hydrogelation at 37°C. Cells were expected to distribute homogeneous and grow in 3D within the hydrogel architecture. To examine cell distribution, 5-day-incubated cell clusters in hydrogel were stained with a nucleic acid fluorescent dye (Hoechst 33342), encapsulated in h9e/MEM hydrogel, and observed using a LSCM with z-stack imaging. The homogeneous cells distribution was proved in both orthotropic and 3D view of LSCM z-stacks images (Figure 3A), indicating that the peptide assembly into a nanofiber network is fast, and the gel is strong enough to suspend the cells. Cell clusters in round shapes were observed in the XY axis view of the orthotropic image (Figure 3A), and multiple nuclei were presented in each cluster meaning that each cluster consists of many cells. This phenomenon was further confirmed in the inverted microscope image of MCF-7 cells in 3D hydrogel (Figure 3B). The tumor-like clusters formation was observed, suggesting that a single seeded cell can form 3D cluster in hydrogel after multiple cell divisions. In contrast, the MCF-7 cells cultured in 2D environment attached to the bottom of the plastic plate and presented a flat shaped morphology (Figure 3C).

**Figure 4:**
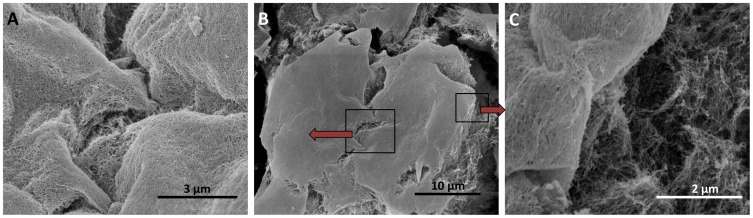
SEM images of cell cluster in 3D hydrogel matrix. **A.**Nanofiber network coating on cell surface. **B.** A protruding cell cluster from the nanofiber scaffold. **C.** Interface of the cell surface and the surrounding hydrogel matrix.

SEM images reveal the interaction of cells and the surrounding hydrogel matrix (Figure 4). Figure 4B is a protruding cell cluster from the nanofiber scaffold. Mutiple layers of nanofiber network attaching on the cell surface (Figure 4A) indicates that the 3D cell cluster was formed by multiple cell divisions of a single seeded cell. The expanding cells force the surrounding hydrogel matrix to collapse into layers of nanofiber network and attach to the cell surface. Such interface character of the cell surface and the surrounding hydrogel matrix (Figure 4C) suggests that MCF-7 cells in 3D culture were supported by the hydrogel matrix. The hydrogel matrix provides a scaffold environment on which cells could stably settle, but from which they also are able to extrude into three dimensions when they divide.

### Cell Isolation from the Hydrogel Matrix

**Figure 5:**
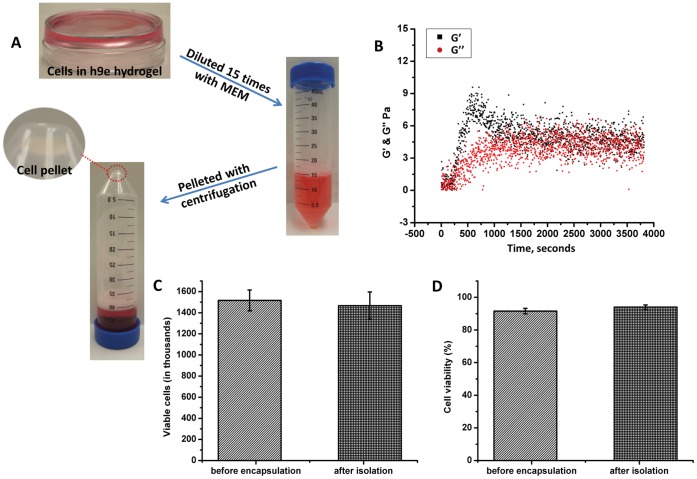
Cell isolation from hydrogel matrix. **A.** Cells encapsulating peptide hydrogel diluted in MEM and cell pelleted with 5-minute centrifugation at 4°C. **B.** Storage G′ and loss G″ moduli hydrogel diluted 15 times at 1 Hz frequency and 1% shear strain at 4°C for 1 hour. **C.** Viable cells before cell encapsulation and after cell isolation. **D.** Cell viability before cell encapsulation and after cell isolation. *p<0.05, n = 3, error bars represent standard deviation.

Isolation of cultured cells from the hydrogel matrix becomes a critical step for further cell physiological and pathophysiological studies. According to the rheological study above, hydrogel architecture can be destroyed under shear stress. Therefore, the hydrogel matrix can be converted to liquid by simply shearing procedure. As mentioned before, the fast reassembly property of this hydrogel is correlated to the peptide concentration. To assure enough time for cell isolation, the sheared hydrogel with 3D cultured cells need to be diluted and maintained at a low environmental temperature. In practice, a 3 mM peptide hydrogel was diluted 15 times with MEM and thoroughly mixed using a pipette, yielding a finial peptide concentration of 0.2 mM (Figure 5A). The cells/h9e peptide/MEM mixture was converted to a liquid form. After 5 minutes of centrifugation at 4°C, the cells were clearly separated from the h9e peptide/MEM solution into a small pellet, which was observed at the bottom of the centrifuge tube (Figure 5A). To confirm that hydrogel solution is in low-viscosity liquid form during the isolation, after dilution and pipetting, the G′ and G″ of this solution were determined by a 1 Hz single frequency rheological study with 1% shear strain. The experimental temperature was set at 4°C, the same as that used for cell centrifugation. Figure 5B indicates that after dilutions, the hydrogel was converted to Newtonian liquid showing that both G′ and G″ were around 0 Pa for the first 200 seconds. The G′ then increased to around 9 Pa within the first 10 minutes; however, it quickly dropped to 5 Pa and stayed at the same level as G″ for the remaining test. This result suggests that although the peptide has the tendency to reassemble, its solid strength is too weak to resist a slight disturbance, even shear stress from 1% strain. The diluted solution become stable as a low-viscosity liquid and would not reform as a hydrogel within the time domain for centrifuge isolation process (Figure 5B). Besides, the shear stress encountered during centrifugation is much higher than 1% shear strain, which keeps the hydrogel solution in low-viscosity liquid form and makes cell isolation from this solution even easier.

H9e peptide hydrogel provides a convenient method of isolating cells from the 3D hydrogel matrix. The next question is what percentage of cells in the hydrogel matrix could be collected from this method and whether they are viable after extraction. To address this question, 1.5×10^5^ cells were seeded in 3 mM h9e hydrogel and isolated using the above method after 30 minutes of incubation. Cell viability before encapsulation and after isolation was compared (Figure 5C, D). No significant difference was found in viable cell number and cell viability before encapsulation and after isolation (Figure 5C, D). These results suggest that cell isolation method for the h9e peptide hydrogel is safe and effective for biological studies.

### Comparisons of Cell Morphology, Viability, and Proliferation in 2D Monolayer and 3D h9e Hydrogel

**Figure 6:**
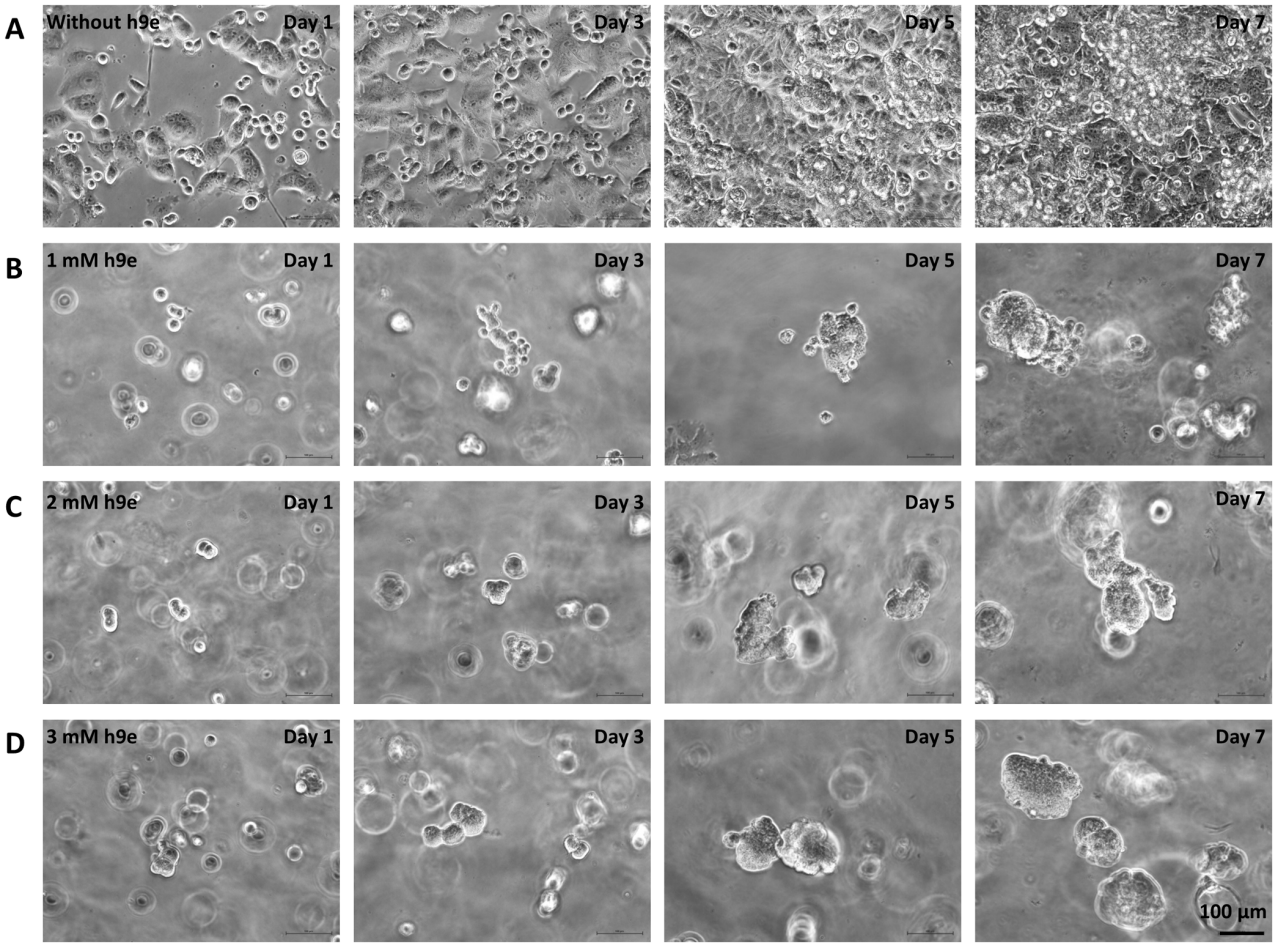
Cell morphologies at days 1, 3, 5, and 7 in 2D plate and 3D hydrogels. **A.** Microscope images of cell growth on 2D monolayer over 7 days. **B.** Microscope images of cell growth in 1 mM h9e peptide of 3D hydrogel over 7 days. **C.** Microscope images of cell growth in 2 mM h9e peptide of 3D hydrogel over 7 days. **D.** Microscope images of cell growth in 3 mM h9e peptide of 3D hydrogel over 7 days.

Cells were cultured in 2D monolayer (without h9e peptide) and in 1–3 mM 3D h9e hydrogel matrices for 1, 3, 5, and 7 days. Differences in cell morphology were first observed under 20X magnification of light microscopy. The morphological observation shows that, after the first 24-hour incubation, cells in the 2D plate attached to the the surface of plastic flask as shown in Day 1 of Figure 6A. Cells in the 3D architectures were floating in the gel matrix with a similar sphere shape in all three peptide hydrogel concentrations (Day 1 of Figure 6B–D). Over 3 days, cells in the 2D plate stretched as a monolayer; by day 5, without any attaching space on the plastic bottom, cells started to grow on top of the attaching layer and kept spreading and overlaying each other by day 7 (Day 3–7 of Figure 6A). In contrast, cells in the 3D matrix grew as rounded clusters over time. Compared with cells in 2 and 3 mM peptide hydrogel, which compacted more tightly and showed a cluster with multiple nuclei, some individual cells were localized on the edge of larger clusters of cells in 1 mM peptide hydrogel at days 3 and 5 (Day 3 and 5 of Figure 6B–D). This indicates that cells migrate easier in a loose nanofiber matrix (Figure 1C), but are encapsulated tightly in a highly dense nanofiber network (Figure 1D). In addition to the growth of the individual cluster, the neighboring clusters were merging into larger clusters, and the cells in all 3 concentrations of h9e peptide hydrogels became similar in morphology by day 7 (Day 7 of Figure 6B–D).

**Figure 7:**
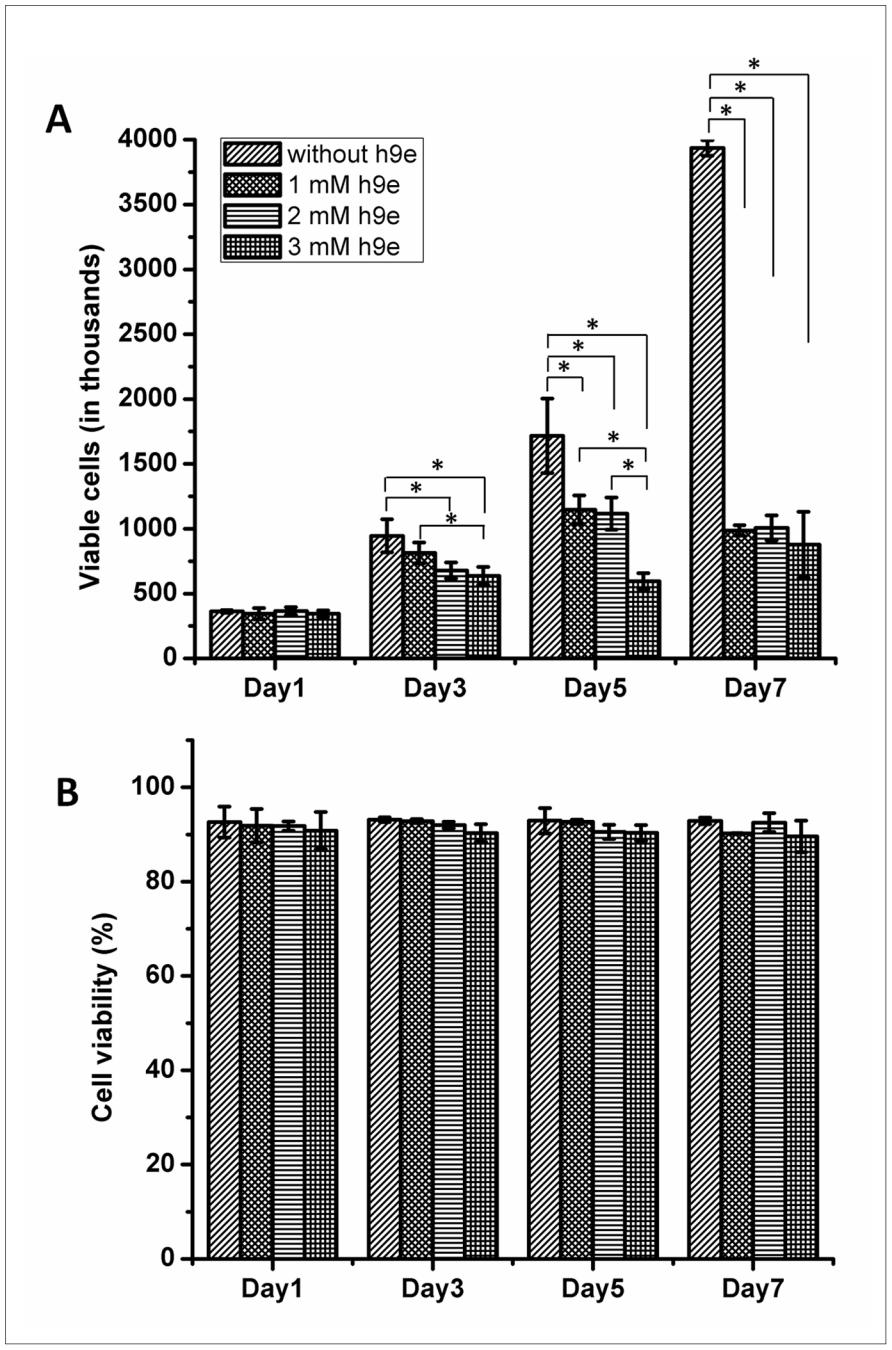
Cell viability at days 1, 3, 5, 7 in 2D monolayer and 3D hydrogel. A. Viable cells of 1, 3, 5, and 7 days cultured in 2D monolayer and 3D hydrogel. **B.** Cell viability at days 1, 3, 5, 7 in 2D monolayer and 3D hydrogel. *p<0.05, n = 3, error bars represent standard deviation.

With the cell isolation capability, the proliferation and viability of MCF-7 cells cultured in the 3D hydrogel matrix were determined. Cell viability was determined using Trypan blue excision method. Cells in the 2D culture proliferate rapidly by doubling in number every other day over 7 days of observation (Figure 7A). Like many reported 3D cell cultures [6], [38], [39], cells in h9e hydrogel have a lower proliferation rate than those in the 2D culture (Figure 7A). Cells in 1 mM hydrogel proliferate faster than those in 3 mM hydrogel by day 3, which may be due to the cell migration providing more space for cell division. By day 5, cells in both 1 and 2 mM hydrogel matrix had similar cell numbers and remained in a steady state by day 7 (Figure 7A). Cells in 3 mM peptide hydrogel proliferate even slower and remained in a relatively steady state from day 3. Cell proliferation rate of 2D and 3D cultures with various concentrations of h9e peptide has shown to be different. Interestingly, cell viability with various concentrations of h9e peptide in 2D or 3D cell culture stayed constant (Figure 7B).

### Effects of Cisplatin, an Anti-cancer Drug, on MCF-7 Cells in 3D h9e Hydrogel

**Figure 8:**
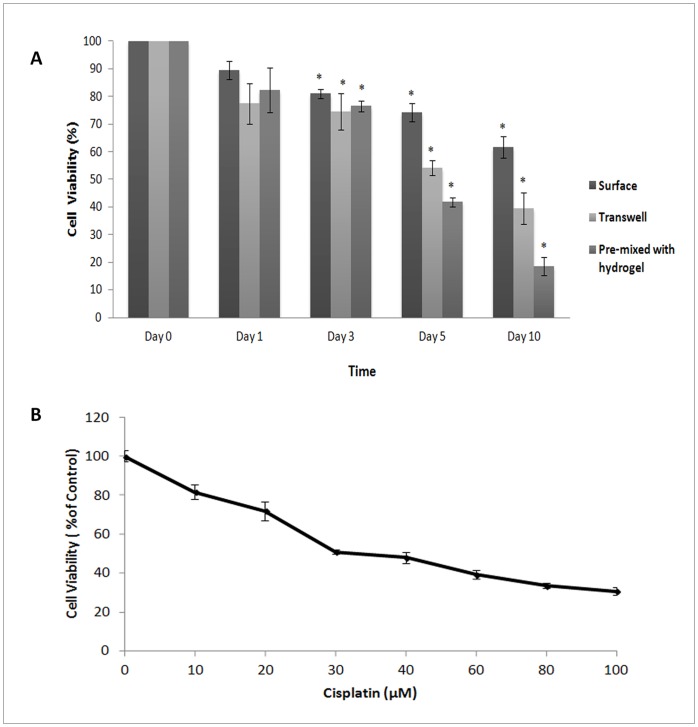
Effect of ciplatin on cell viability via three methods of treatment and IC_50_ for cisplatin in h9e hydrogel culture. **A**. Cells were cultured in hydrogel for 5 days and were treated with 40 µM cisplatin for 0, 1, 3, 5, and 10 days via three methods:first, cells were grown in hydrogel and dosed with cisplatin by placing medium with cisplatin on the top surface of the hydrogel; second,cells were grown in hydrogel on a transwell insert, and medium with cisplatin was placed on top and bottom of the transwell insert; third, ciplatin was mixed with hydrogel and cells prior to hydrogelation. Cell viability was measured using Trypan blue excision method. Data were obtained in three independent experiments and are represented as the mean ± S.D. * P-value is <0.05 compared to control of cells without treatment at appropriate time point. **B.** MCF-7 cells were cultured in hydrogel for 5 days. After 5 days of colony formation, cells were harvested and treated with various concentrations of cisplatin for additional 5 days in hydrogel culture using “pre-mixed“ method as previously described. Cells were harvested and performed viability assay using Trypan blue excision method. Data were normalized to the control of cells without treatment. Data were obtained in three independent experiments and are represented as the mean ± S.D. The results shows that IC_50_ of cisplatin in MCF-7 cells under hydrogel culture is 31.25 µM.

The application of the h9e hydrogel as a 3D cell culture system for drug testing was examined using anti-cancer drug, cisplatin. To test drug diffusion in hydrogel, 5-day 3D cultures were treated with 40 µM cisplatin via three methods as described in *Materials and Methods*: top surface, top-bottom transwell, and pre-mixed with medium and h9e peptide prior to hydrogelation. The results show that cisplatin can cause a decrease of cell viability through all three methods, indicating that all the methods can deliver cisplatin throughout the hydrogel matrix (Figure 8A). At Day 3 after treatment, a significant 20% decrease of cell viability is observed in cells treated with 40 µM cisplatin via all the methods (Figure 8A). However, at Day 10, 40 µM cisplatin can cause 40%, 60% and 85% decrease in cell viability via top surface, top-bottom transwell and pre-mixed with hydrogel method, respectively (Figure8A), suggesting that cisplatin is more effective when pre-mixed with hydrogel. All the results demonstrate that h9e peptide hydrogel permits the nutrients and drugs to diffuse throughout the matrix.

Figure 8A shows cell responding time to cisplatin treatment in 3D hydrogel. Furthermore, cultured cells in hydrogel was tested with multiple doses of cisplatin. Cells were cultured in hydrogels for 5 days to form tumor-like clusters, and then treated with cisplatin via pre-mixed method as previously described. Cells were treated for 5 days and the IC_50_ of cisplatin was determined. The results show that cisplatin decreases the viablity of cells in hydrogel in a dose-dependent manner (Figure 8B). The IC_50_ of cisplatin in MCF-7 cells under hydrogel condition is 31.25 µM (Figure 8B), which is consistent with the literature for cisplatin, suggesting that hydrogel has the appropriate matrix for drug testing [40].

**Figure 9:**
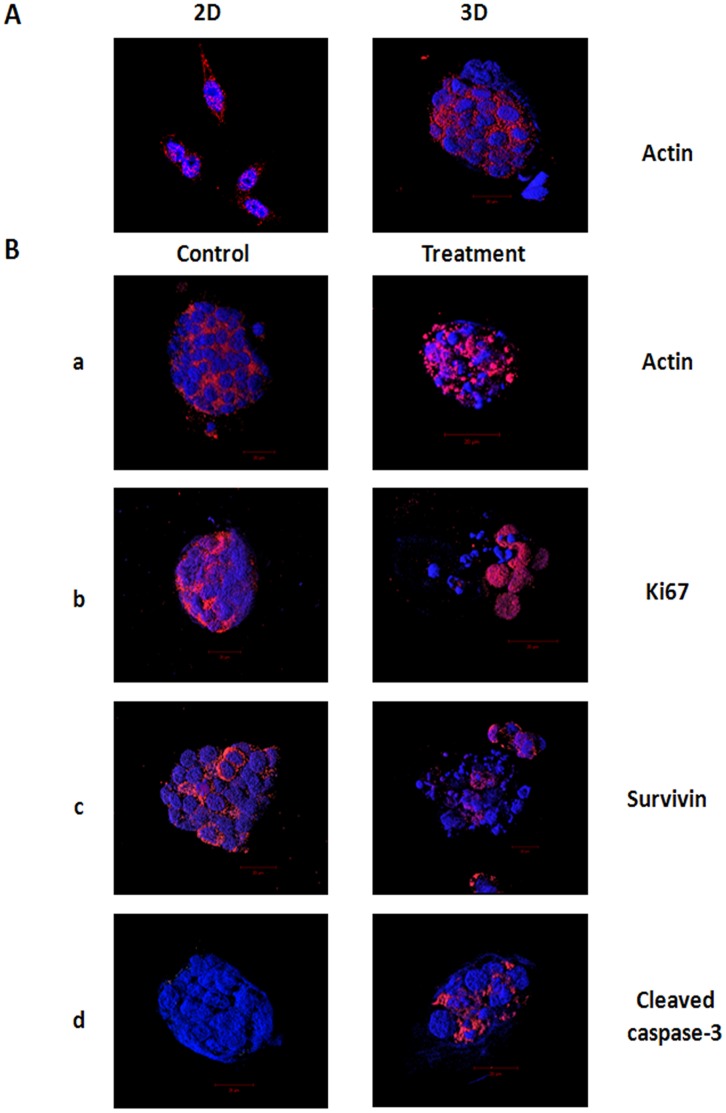
Immunofluorescence assay of cells in 3D hydrogel. **A.** MCF-7 cells were cultured in 2D monolayer for 2 days **(2D)** or in 3D hydrogels for 7 days **(3D)**. Immunofluorescence assay was performed. Red indicates actin and blue indicates DAPI-stained nuclei. **B.** MCF-7 cells were cultured in hydrogel for 5 days. After 5 days of colony formation, cells were cultured in 3D hydrogel for additional 2 days **(Control)** or treated with 30 µM cisplatin for 48 hours in hydrogel via “pre-mixed” method **(Treatment)**. Cells were isolated from hydrogels and immunostained with antibodies against actin (**a**), Ki67 (**b**), survivin (**c**), and cleaved caspase-3 (**d**). Red indicates protein of interest and blue indicates DAPI-stained nuclei. To obtain the 3D images, Z-stack images were taken (0.5–1 µm slices) and reconstituted in ZEN 2010 software. The results represent one of three independent experiments.

The effects of cisplatin on cells in h9e hydrogel was further studied using confocal microscopy assay. Cells were cultured in 3 mM hydrogel for 5 days and then treated with 30 µM cisplatin for 48 hours via pre-mixed method. Seven-day cell cultures in hydrogel matrix without treatments were used as controls. After treatment, cells were isolated from the hydrogel and immunostained with antibodies against actin, Ki67, survivin, and cleaved caspase-3. DAPI was used to stain nuclei. Images of cells were captured with confocal fluorescence microscope (Carl Zeiss LSM 700 META). To obtain the 3D images, Z-stack images were taken (0.5–1 µm slices) and reconstituted in ZEN 2010 software. In this study, the morphology of 3D cell clusters in hydrogel matrix was first obseved by comparing to cells cultrued in 2D (Figure 9A). Consistent with previous results (Figure 6D), 7-day culture in h9e hydrogel allows MCF-7 cells to form tumor-like clusters with the diameter of >50 µm (Figure 9A). The cells in the hydrogel displays a rounded, clustered morphology distinct from the spread morphology of the cell in 2D monolayer (Figure 9A). Immunofluorescence stain against actin showed to be localized in the cytoplatsmic region in both 2D and 3D cultures; however, unlike the elongated expression of actin in 2D cell culture, actin in 3D cell culture was expressed around the parimeter of the cell (Figure 9A). Compared to the 3D clusters without cisplatin treatment, nuclear fragmentation was observed in all cisplatin-treated clusters (Figure 9B). Actin was used as a biomarker in examing the effect of cisplatin on cytoskeleton. In the cisplatin-untreated clusters, actin was detected around the cell perimeter with an intact organization (Figure 9B-a). However, disruption of spheroids was observed in cisplatin-treated samples (Figure 9B-a). Ki67, a proliferation biomarker, was highly expressed in the control spheroids (Figure 9B-b), suggesting that cells cultured in hydrogel have proliferative ability. In the cisplatin-treated spheroids, Ki67 staining was only observed in cells with unchanged nuclei (Figure 9B-b). Furthermore, the expressions of survivin, an inhibitor of apoptosis, and cleaved caspase-3, an activator of apoptosis were selected to examine the effect of cisplatin on apoptosis of 3D cell cultures in hydrogel. The expression of survivin was only detectd around very fewintact nuclei (Figure 9B-c), and cleaved caspase-3 was highly expressed in the cisplatin-treated spheroids (Figure 9B-d), indiating that cisplatin can induce apoptosis in cells cultured in hydrogel. Overall, the confocal microscopy results provide evidence that cisplatin can diffuse into h9e hydrogel and exert its effects on cytoskeleton structure, proliferation, and apoptosis of MCF-7 cells in 3D hydrogel culture.

**Figure 10:**
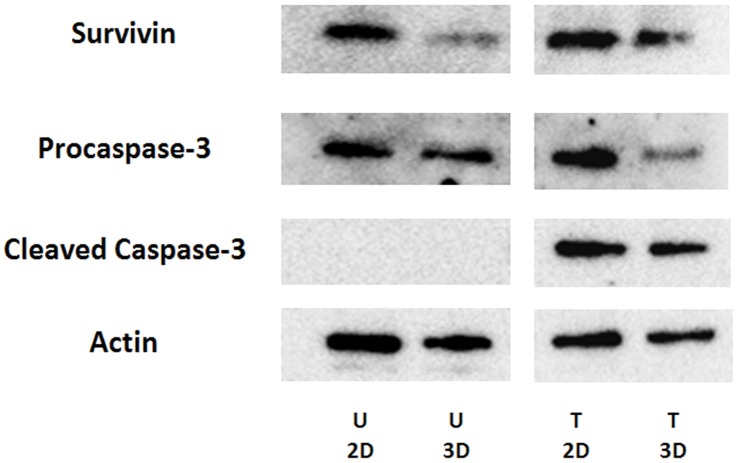
Western blot analysis of cells in 2D monolayer and 3D hydrogel. MCF-7 cells were cultured in 2D monolayer and 3D hydrogel for 7 days **(U)** or for 5 days and then treated with 30 µM cisplatin for 48 hours **(T)**. Cells were harvested and whole cell extracts were obtained. Western blot analysis of survivin, procaspase-3, and cleaved caspase-3 was performed. Actin was used as a loading control. The results represent one of three independent experiments.

The ability to recovery cells from 3D culture is an important features of the h9e hydrogel for cell culture and drug testing. Recoved cells from the 3D matrix are critical for further analysis. To examine this unique property of h9e hydrogel, Western blot analysis was performed. MCF-7 cells were cultured in 2D monolayer and 3D hydrogels for 7 days without any treatments (Figure 10 untreated), or treated with 30 µM cisplatin for 48 hours after 5-day culture (Figure 10 treated). For the 3D clusters in hydrogel, cisplatin was added via pre-mixed method. Cells were harvested from 2D culture or isolated from the hydrogels and whole cell extracts were obtained for Western blot analysis. The expressions of apoptotic markers (survivin, procaspase-3 and cleaved caspase-3) were detected. The results showed that in untreated cells, expressions of survivin and procaspase-3 were detected in both 3D and 2D culture; however, no cleaved caspase-3 was detected in either 2D or 3D cultures through Western blot analysis (Figure 10 untreated). Because cleavage of caspase-3 is a hallmark of apoptosis, this result indicates that the h9e peptide hydrogel is an advanced material for *in vitro* 3D cell culture without induction of apoptosis. Consistent with previous confocal imaging results (Figure 9B), all markers were dectected in 3D clusters treated with cisplatin (Figure10 treated). These results indicate that cells cultured and treated in h9e peptide hydrogel system can be extracted and analyzed for the downstream proteomic analysis.

### Conclusions

In this study, a self-assembly peptide hydrogel system for 3D cell culture was examined. MCF-7 cells were encapsulated homogeneously during the hydrogelation simply by adding peptide solution to cell suspension of MEM. At physiological pH, the formation of hydrogel with controlled time and gel strength can be modulated by changing the temperature or peptide concentration. The specific shear thinning and rapid recovery rheological properties of hydrogel allow us to deliver this hydrogel material via pipetting multiple times. Unlike the attached flat morphology of cell in a 2D monolayer culture, cells residing in h9e peptide hydrogel adopt clustered structures that are reminiscent of tumor-like structure. More advantageously, the hydrogel architecture can be disrupted by pipetting mixing and further converted into liquid form through dilution with MEM medium. This property provides a convenient and safe method to isolate 3D cultured cells effectively from the hydrogel matrix by centrifugation. Compared with the 2D system, the cell proliferation rate is much slower in the 3D system and relative to different concentrations of h9e peptide. Cells cultured in 3D hydrogel culture have high viability and no significant apoptosis is induced by the 3D culture. The anti-cancer drug, cisplatin, can diffuse throughout the h9e hydrogel matrix and the h9e hydrogel allows testing of cisplatin in a time- and dose- dependent manner. Cells can be recovered from the 3D hydrogel system and used for the proteomic analysis, targeting specific pathways of drug-mediated response. Overall, h9e peptide hydrogel provides a promising 3D cell culture system for drug testing and other biomedical applications.

## Supporting Information

Table S1 **Comparison of material properties, cell encapsulation/recovery, and handling of different 3D cell culture hydrogels.**
(DOCX)
