# Supplementary material for: Peptide Hydrogelation and Cell Encapsulation for 3D Culture of MCF-7 Breast Cancer Cells
Source: PLoS One. 2013 Mar 20;8(3):e59482. doi: 10.1371/journal.pone.0059482 (PMC3603912; doi:10.1371/journal.pone.0059482)
Supplement: Table S1 — Comparison of material properties, cell encapsulation/recovery, and handling of different 3D cell culture hydrogels. (DOCX) [file pone.0059482.s001.docx]

**Table S1. Comparison of material properties, cell encapsulation/recovery, and handling of different 3D cell culture hydrogels**

| Characteristics | h9e | Puramatrix gel (BD Biosciences) [a] | Matrigel (BD Biosciences) [b] | Alginate hydrogel (ALgimatrix) [c] |
| --- | --- | --- | --- | --- |
| Material | Peptide (19 unit) | peptide (16 unit) | Reconstituted basement membrane extracted from EHS mouse tumor | Polysaccharides  (dried sponge) |
| Porositys | 50-200 nm | 50-200 nm | 50-400 nm | 50-200 μm |
| Solution pH | Neutral | Acidic pH 3 | Various during the storage (acidic to physiological pH) | Dry |
| Gel trigger | Hydrogel could be triggered by directly mixing cell medium or solution containing Ca^2+^, Na^+^ (no pH or temperature adjustment) | Starts gel at pH higher than 4.5-5 (change medium at least 3 steps within first 30 min to equilibrate the sample to physiological pH). | Starts gel at temperature higher than 10 ℃ | Add gel firming buffer containing Ca^2+^ |
| Cell encapsulation | Directly mix (pipette), cells suspended in cell medium before the peptide solution is added in a relaxed working environment. Cells are surrounded by medium and nanofibrils network during hydrogelation. | Directly mix (pipette, has to be very fast, within 1 min, to shorten the contact time of cell with acidic peptide solution); cells is isolated from medium and prepared in 10% sucrose solution before peptide solution is added | Directly mix with chilled pipette (need to chill everything before experiment because temperature is the trigger for gelation) | Immediately centrifuge after the firming buffer added (for better cell distribution) |
| Cell recovery | Pipette, dilute the hydrogel with cell medium 1:15 folds and centrifuge | Pipette to disturb the gel structure and centrifuge | Add cell recovery solution or lowing temperature or centrifugation to disrupt the gel matrix | Add dissolving buffer |

[a][b][c] cited from company’s website
